# Evaluation of the efficacy and safety of recombinant erythropoietin on the improvement of hospitalised COVID-19 patients: A structured summary of a study protocol for a randomised controlled trial

**DOI:** 10.1186/s13063-021-05363-w

**Published:** 2021-07-06

**Authors:** Hamid Reza Samimagham, Mehdi Hassani Azad, Dariush Hooshyar, Maryam Haddad, Mohsen Arabi, Mitra KazemiJahromi

**Affiliations:** 1 Clinical Research Development Center, Shahid Mohammadi Hospital, Hormozgan University of Medical Sciences, Bandar Abbas, Iran; 2 Infectious and Tropical Diseases Research Center, Hormozgan Health Institute, Hormozgan University of Medical Sciences, Bandar Abbas, Iran; 3 Student Research Committee, Faculty of Medicine, Hormozgan University of Medical Sciences, Bandar Abbas, Iran; 4 Department of Internal Medicine and Public Health Research Center, Family Medicine Department, Iran University of Medical Sciences, Tehran, Iran; 5 Endocrinology and Metabolism Research Center, Hormozgan University of Medical Sciences, Bandar Abbas, Iran

**Keywords:** COVID-19, Randomised controlled trial, protocol, recombinant erythropoietin, erythropoietin, Phase 2

## Abstract

**Objectives:**

To evaluate the effect of recombinant erythropoietin on hospitalised COVID-19 patients.

**Trial design:**

Concealed, randomized, single-blinded, phase 2 controlled clinical trial with two arm parallel-group design of 20 patients allocated with 1:1 ratio and using the placebo in the control group.

**Participants:**

This study will be performed at Shahid Mohammadi Hospital in Bandar Abbas, Hormozgan in Iran. All positive (PCR confirmed) COVID-19 patients ≤65 years old who have Hb≤9 and at least one of the severe COVID-19 symptoms (tachypnea (breathing rate> 30 beats per minute), hypoxemia (O2 ≤93 saturation, the partial pressure ratio of arterial oxygen <300), Lung infiltration (> 50% of lung field within 24 to 48 hours), progressive lymphopenia, LDH>245 U/I, CRP>100) and are willing to cooperate in this project will be included in the study. Patients with a history of coronary heart disease, thrombosis, deep vein thrombosis, chronic lung disease, diabetes mellitus, weakened immune system, end-stage renal disease, liver disease, and patients with a history of taking oral contraceptive pills, systolic blood pressure more than 160 mm Hg, diastolic blood pressure more than 90 mm Hg and age over 65 and erythropoietin above 500 are excluded.

**Intervention and comparator:**

Patients will receive the standard of care (SOC) based on the treatment protocols of the Iranian National Committee of COVID-19 and recombinant erythropoietin (EPREX Manufactured by Johnson and Johnson Pharmaceutical Company) 300 units / Kg or 4000IU as subcutaneous (SQ) injection three times a day for 5 days and simultaneously Enoxaparin 1 mg/kg SQ daily is also taken to prevent thrombosis in the intervention group. Patients' blood pressure, along with other vital signs, are checked regularly and at regular intervals.

In the control group, patients received SOC and the placebo (distilled water) is given as a subcutaneous injection three times a day for 5 days. We use sterile water for injection (EXIRpharmaceutical company) as the placebo. To the same appearance of the placebo and the recombinant erythropoietin, they are taken in a separate room in the same size syringes and cover with labels before injection.

**Main outcomes:**

The main outcome for this study is a composite endpoint for Patient clinical symptoms (Respiratory rate, Oxygen saturation state and arterial oxygen partial pressure ratio, Lung infiltration status, blood pressure), Laboratory tests (LDH, CRP, Lymphocyte count, Endogenous erythropoietin, and Haemoglobin level). All of these will be assessed at the beginning of the study (before the intervention) and day 5 after the intervention. The study will also evaluate side effects and how to manage them.

**Randomisation:**

Eligible participants (20) will be randomized in two arms in the ratio of 1: 1 (10 per arm) by permuted block randomization method using online web-based tools.

**Blinding (masking):**

Patients participating in the study will not be aware of the assignment to the intervention or control group. The principal investigator, health care personnel, data collectors, and those evaluating the outcome are aware of patient grouping.

**Numbers to be randomised (sample size):**

A total of 20 patients will participate in this study, who are randomly allocated to the 2 arms with a 1:1 ratio; 10 patients in the intervention group will receive SOC and recombinant erythropoietin, and 10 patients in the control group will receive SOC and placebo.

**Trial Status:**

The protocol version is 3.0, approved by the Deputy of Research and Technology and the ethics committee of Hormozgan University of Medical Sciences on 6^th^ June 2020, with the local grant number of 990108. The expected recruitment end date was on 21^th^ December 2020 but since we had a wide and careful exclusion criteria because of the adverse reactions of the medication, the recruitment (for both cases and controls) was not so easy and did not finish on the expected date and we are still recruiting now. Recruitment began on 17^th^ August 2020 and the updated expected recruitment end date is 1^st^ August 2021.

**Trial registration:**

The protocol was registered before starting subject recruitment under the title: Evaluation of the effect of recombinant erythropoietin on the improvement of COVID-19 patients, IRCT20200509047364N1, at Iranian Registry of clinical trials (https://en.irct.ir/trial/49282) on 2020/08/09.

**Full protocol:**

The full protocol is attached as an additional file, accessible from the Trials website (Additional file 1). In the interest in expediting dissemination of this material, the familiar formatting has been eliminated; this Letter serves as a summary of the key elements of the full protocol.

The study protocol has been reported in accordance with the Standard Protocol Items: Recommendations for Clinical Interventional Trials (SPIRIT) guidelines (Additional file 2).

**Supplementary Information:**

The online version contains supplementary material available at 10.1186/s13063-021-05363-w.

## Supplementary Information

**Additional file 1.** Full study protocol.

**Additional file 2.** SPIRIT 2013 Checklist: Recommended items to address in a clinical trial protocol and related documents*.

## Data Availability

The researchers in this study have not yet made a comprehensive decision to share information.

