# Supplementary material for: Evaluation of the efficacy and safety of recombinant erythropoietin on the improvement of hospitalised COVID-19 patients: A structured summary of a study protocol for a randomised controlled trial
Source: Trials. 2021 Jul 6;22:435. doi: 10.1186/s13063-021-05363-w (PMC8258474; doi:10.1186/s13063-021-05363-w)
Supplement: Supplementary file 1 — Additional file 1. Full study protocol. [file 13063_2021_5363_MOESM1_ESM.pdf]

Letter to the editor:

Title
Evaluation of the efficacy and safety of recombinant erythropoietin on the improvement of hospitalised COVID-19 patients: A structured summary of a study protocol for a randomised controlled trial

Authors
Hamid Reza Samimagham[1], Mehdi Hassani Azad[2], Dariush Hooshyar[3], Maryam Haddad[1], Mohsen Arabi[4], Mitra KazemiJahromi[5]*

[1]Clinical Research Development Center, Shahid Mohammadi Hospital, Hormozgan University of Medical Sciences, Bandar Abbas, Iran
[2]Infectious and Tropical Diseases Research Center, Hormozgan Health Institute, Hormozgan University of Medical Sciences, Bandar Abbas, Iran
[3]Student Research Committee, Faculty of Medicine, Hormozgan University of Medical Sciences, Bandar Abbas, Iran
[4]Department of Internal Medicine and Public Health Research Center, Family Medicine Department, Iran University of Medical Sciences, Tehran, Iran
[5]Endocrinology and Metabolism Research Center, Hormozgan University of Medical Sciences, Bandar Abbas, Iran

Email: samimagham@yahoo.com[1], mehdihassaniazad@gmail.com[2], dariush.hooshyar@gmail.com[3] , Dr.maryhd@gmail.com [1], arabi.m@iums.ac.ir[4], mitra.kazemijahromi@gmail.com [5],*

Correspondence to*: Mitra KazemiJahromi[5]*
Tel: +00989177912820*

Abstract
Objectives
To evaluate the effect of recombinant erythropoietin on hospitalised COVID-19 patients.
Trial design
Concealed, randomized, single-blinded, phase 2 controlled clinical trial with two arm parallel-group design of 20 patients allocated with 1:1 ratio and using the placebo in the control group.
Participants
This study will be performed at Shahid Mohammadi Hospital in Bandar Abbas, Hormozgan in Iran.All positive (PCR confirmed) COVID-19 patients ≤65 years old who have Hb≤9 and at least one of the severe COVID-19 symptoms (tachypnea (breathing rate> 30 beats per minute), hypoxemia (O2 ≤93 saturation, the partial pressure ratio of arterial oxygen <300), Lung infiltration (> 50% of lung field within 24 to 48 hours), progressive lymphopenia, LDH>245 U/l, CRP>100) and are willing to cooperate in this project will be included in the study. Patients with a history of coronary heart disease, thrombosis, deep vein thrombosis, chronic lung disease, diabetes mellitus, weakened immune system, end-stage renal disease, liver disease, and patients with a history of taking oral contraceptive pills, systolic blood pressure more than 160 mm Hg, diastolic blood pressure more than 90 mm Hg and age over 65 and erythropoietin above 500 are excluded.

Intervention and comparator
Patients will receive the standard of care (SOC) based on the treatment protocols of the Iranian National Committee of COVID-19 and recombinant erythropoietin (EPREX Manufactured by Johnson and Johnson Pharmaceutical Company) 300 units / Kg or 4000IU as subcutaneous (SQ) injection three times a day for 5 days and simultaneously Enoxaparin 1 mg/kg SQ daily is also taken to prevent thrombosis in the intervention group. Patients' blood pressure, along with other vital signs, are checked regularly and at regular intervals. In the control group, patients received SOC and the placebo (distilled water) is given as a subcutaneous injection three times a day for 5 days We use sterile water for injection (EXIR pharmaceutical company) as the placebo. To the same appearance of the placebo and the recombinant erythropoietin, they are taken in a separate room in the same size syringes and cover with labels before injection.

Main outcomes
The main outcome for this study is a composite endpoint for Patient clinical symptoms(Respiratory rate, Oxygen saturation state and arterial oxygen partial pressure ratio, Lung infiltration status, blood pressure), Laboratory tests (LDH, CRP, Lymphocyte count, Endogenous erythropoietin, and Haemoglobin level). All of these will be assessed at the beginning of the study (before the intervention) and day 5 after the intervention. The study will also evaluate side effects and how to manage them.

Randomisation
Eligible participants (20) will be randomized in two arms in the ratio of 1: 1 (10 per arm) by permuted block randomization method using online web-based tools.**Blinding (masking)**
Patients participating in the study will not be aware of the assignment to the intervention or control group. The principal investigator, health care personnel, data collectors, and those evaluating the outcome are aware of patient grouping.

Numbers to be randomised (sample size)
A total of 20 patients will participate in this study, who are randomly allocated to the 2 arms with a 1:1 ratio; 10 patients in the intervention group will receive SOC and recombinant erythropoietin, and 10 patients in the control group will receive SOC and placebo.

Trial Status
The protocol version is 3.0, approved by the Deputy of Research and Technology and the ethics committee of Hormozgan University of Medical Sciences on 6th June 2020, with the local grant number of 990108. The expected recruitment end date was on 21th December 2020 but since we had a wide and careful exclusion criteria because of the adverse reactions of the medication, the recruitment (for both cases and controls) was not so easy and did not finish on the expected date and we are still recruiting now. Recruitment began on 17th August 2020 and the updated expected recruitment end date is 1st August 2021.

Trial registration
The protocol was registered before starting subject recruitment under the title: Evaluation of the effect of recombinant erythropoietin on the improvement of COVID-19 patients, IRCT20200509047364N1, at Iranian Registry of clinical trials (https://en.irct.ir/trial/49282) on 2020/08/09.

Full protocol
The full protocol is attached as an additional file, accessible from the Trials website (Additional file 1). In the interest in expediting dissemination of this material, the familiar formatting has been eliminated; this Letter serves as a summary of the key elements of the full protocol.
The study protocol has been reported in accordance with the Standard Protocol Items: Recommendations for Clinical Interventional Trials (SPIRIT) guidelines (Additional file 2).

Keywords
COVID-19, Randomised controlled trial, protocol, recombinant erythropoietin, erythropoietin Phase 2.

Declarations

Ethics approval and consent to participate
The protocol was approved by the ethics committee of Hormozgan University of Medical Sciences on 2020/06/06, with the code IR.HUMS.REC.1399.165.
The authors authenticate that this trial has received ethical approval from the appropriate ethical committee as described above. Written prospective informed consent will be obtained from participants before involvement in the trial in the Persian language.
Consent for publication
Not applicable
Availability of data and materials
The researchers in this study have not yet made a comprehensive decision to share information.
Competing interests
The authors declare that they have no competing interests
Funding
This trial is supported by the Deputy of Research and Technology of Hormozgan University of Medical Sciences. This sponsor and founder has no role in designing the study, collection, storage, and analysis of information and in preparing the manuscript.
Authors' contributions
MKJ is the Chief Investigator; she conceived the study, led the proposal and protocol development. HRS contributed to study design and to development of the proposal. MA was the lead trial methodologist. DH collaborated in designing the study and writing the reports. MHA, MH, MA, have contributed to the data collection. All authors read and approved the final manuscript.
Acknowledgements
We are sincerely thankful to the Clinical Research Development Center of Shahid Mohammadi Hospital, Bandar Abbas, Iran for its support.
Authors' information (optional)

Clinical Research Development Center, Shahid Mohammadi Hospital, Hormozgan University of Medical Sciences, Bandar Abbas, Iran
Hamid Reza Samimagham and Maryam Haddad

Infectious and Tropical Diseases Research Center, Hormozgan Health Institute, Hormozgan University of Medical Sciences, Bandar Abbas, Iran
Mehdi Hassani Azad

Student Research Committee, Faculty of Medicine, Hormozgan University of Medical Sciences, Bandar Abbas, Iran
Dariush Hooshyar

Department of Internal Medicine and Public Health Research Center, Family Medicine Department, Iran University of Medical Sciences, Tehran, Iran
Mohsen Arabi

Endocrinology and Metabolism Research Center, Hormozgan University of Medical Sciences, Bandar Abbas, Iran
Mitra KazemiJahromi

Clinical Trial Protocol
Iranian Registry of Clinical Trials

26 May 2021

Evaluation of the efficacy and safety of recombinant erythropoietin on the improvement of hospitalised COVID-19 patients

Protocol summary

Study aim

This study aims to investigate the effect of recombinant erythropoietin on the recovery process of COVID-19 patients.

Design

concealed, randomized, single blinded, phase 2 controlled clinical trial with two arm parallel group design of 20 patients, using the placebo in the control group.

Settings and conduct

This study will be performed at Shahid Mohammadi Hospital in Bandar Abbas. Patients enter the study after obtaining written consent, but will not know which group they belong to.

Participants/Inclusion and exclusion criteria

All positive COVID-19 patients who have Hb≤9 and at least one of the severe COVID-19 symptoms and are willing to cooperate in this project will be included in the study. Patients with a history of coronary heart disease, thrombosis, deep vein thrombosis, chronic lung disease, diabetes mellitus, weakened immune system, end stage renal disease, liver disease, and patients with a history of taking oral contraceptive pills, systolic blood pressure more than 160 mm Hg, diastolic blood pressure more than 90 mm Hg and age over 65 and erythropoietin above 500 are excluded.

Intervention groups

Patients are divided into two groups. In group A, the standard treatment for COVID-19 patients with recombinant erythropoietin is prescribed. In group B, patients receive standard COVID-19 treatment with the placebo.

Main outcome variables

Patient clinical symptoms, Laboratory examinations of patients and Hemoglobin

General information

Reason for update

The expected recruitment end date was on 2020/12/21 but since we had a wide and careful exclusion criteria because of the adverse reactions of the medication, the recruitment (for both cases and controls) was not so easy and did not finish on the expected date and we are still recruiting now. Recruitment began on 2020/8/17 and the updated expected recruitment end date is 2021/8/1.

Acronym

IRCT registration information

IRCT registration number: **IRCT20200509047364N1**
Registration date: **2020-08-09, 1399/05/19**
Registration timing: **registered_while_recruiting**

Last update: **2021-05-26, 1400/03/05**
Update count: **2**

Registration date

2020-08-09, 1399/05/19

Registrant information

Name

Dariush Hooshyar

Name of organization / entity

Country

Iran (Islamic Republic of)

Phone

+98 71 3670 3778

Email address

dariush.hooshyar@hums.ac.ir

Recruitment status

recruiting

Funding source

Expected recruitment start date

2020-06-21, 1399/04/01

Expected recruitment end date

2021-08-01, 1400/05/10

Actual recruitment start date

empty

Actual recruitment end date

empty

Trial completion date

empty

Scientific title
Evaluation of the efficacy and safety of recombinant erythropoietin on the improvement of hospitalised COVID-19 patients

Public title
Effect of erythropoietin on COVID-19

Purpose
Treatment

Inclusion/Exclusion criteria
Inclusion criteria:
All definitively positive COVID-19 patients with Hb≤9. Having at least one of the severe symptoms of COVID-19, including tachypnea (breathing rate> 30 beats per minute), hypoxemia (O2 ≤93 saturation, the partial pressure ratio of arterial oxygen <300), Lung infiltration (> 50% of lung field within 24 to 48 hours), progressive lymphopenia, LDH>245 U/l, CRP>100
Exclusion criteria:
Patients with a history of coagulopathy Patients with a history of thrombosis Patients with a history of deep vein thrombosis Patients with a history of chronic lung disease Patients with a history of diabetes mellitus Patients with weakened immune systems Patients with a history of end stage renal disease Patients with liver disease Patients with a history of taking oral contraceptive pills (OCPs) Patients with systolic blood pressure greater than 160 mmHg Patients with diastolic blood pressure greater than 90 mmHg Patients over 65 years of age Patients with erythropoietin above 500 Patients with a history of myocardial infarction or unstable angina Patients with a history of malignancy

Age
To **65 years** old

Gender
Both

Phase
2

Groups that have been masked

- Participant

Sample size
Target sample size: **20**

Randomization (investigator's opinion)
Randomized

Randomization description
Before assigning groups to individuals eligible to participate in the study, informed consent is completed for grouping individuals. the person who has no role in admitting patients and assigning patients to random codes preparing random sequences using online tools (https://www.sealedenvelope.com/) and by permuted block randomization method. Individualized random allocation is done in blocks with sizes 2 and 4, and with stratification based on gender. eligibility criteria are monitored by the person responsible for admitting patients. Codes in a random sequence are assigned to patients by the treatment team without knowing that each code is in the intervention or placebo group. Patient codes are then matched to randomly generated sequence information for interventions. (randomization concealment is done by the treatment team without informing the person responsible for admitting patients and the person who prepared the random sequence.)

Blinding (investigator's opinion)
Single blinded

Blinding description
In this study, all participants are aware of participating in this study and enter the study with their consent. All participants are unaware of which group of this study they are in and after grouping patients in the groups, Patients receive recombinant erythropoietin in the treatment group and receive a placebo in the control group. The lead researcher, health care personnel, data collection officials, and those who evaluate the outcome are aware of the grouping of patients. Those who prepare the draft of the article are unaware of the groupings if they do not cooperate in the above cases.

Placebo
Used

Assignment
Parallel

Other design features

Secondary Ids

empty

Ethics committees

1

Ethics committee
Name of ethics committee
Ethics committee of Hormozgan University of Medical Sciences
Street address
Deputy of Research and Technology, Hormozgan University of Medical Sciences, Shahid Chamran Boulevard, Bandar Abbas, Hormozgan, Iran
City
bandar abbas
Province
Hormozgan
Postal code
7916613885

Approval date
2020-06-06, 1399/03/17

Ethics committee reference number
IR.HUMS.REC.1399.165

Health conditions studied

1

Description of health condition studied
Laboratory confirmed COVID-19

ICD-10 code
U07.1

ICD-10 code description
COVID-19, virus identified

Primary outcomes

1

Description
Respiratory rate
Timepoint
At the beginning of the study (before the intervention) and day 5 after the intervention
Method of measurement
Pulse oximeter

2

Description
Oxygen saturation state and arterial oxygen partial pressure ratio
Timepoint
At the beginning of the study (before the intervention) and day 5 after the intervention
Method of measurement
Pulse oximeter

3

Description
Lung infiltration status
Timepoint
At the beginning of the study (before the intervention) and day 5 after the intervention
Method of measurement
chest x-ray

4

Description
LDH level's
Timepoint
At the beginning of the study (before the intervention) and day 5 after the intervention
Method of measurement
pathobiology laboratory

5

Description
CRP level's
Timepoint
At the beginning of the study (before the intervention) and day 5 after the intervention
Method of measurement
pathobiology laboratory

6

Description
Lymphocyte count
Timepoint
At the beginning of the study (before the intervention) and day 5 after the intervention
Method of measurement
pathobiology laboratory

7

Description
Endogenous erythropoietin level's
Timepoint
Beginning of study (before intervention)
Method of measurement
Pathobiology laboratory

8

Description
Patients' blood pressure
Timepoint
At the beginning of the study (before the intervention), during the intervention (5-day erythropoietin administration)
Method of measurement
Sphygmomanometer

Secondary outcomes

1

Description
Patients' blood hemoglobin levels
Timepoint
Start of study (before the intervention), day 5 after the intervention and 2 to 4 weeks after the intervention
Method of measurement
Pathobiology laboratory

Intervention groups

1

Description
Intervention group: Group A.: The standard drug treatment is based on the treatment protocols of the National Committee of COVID-19 and erythropoietin recombinant (EPREX Manufactured by Johnson and Johnson Pharmaceutical Company) 300 units / Kg or 4000IU as subcutaneous injection five times a day for 5 days and simultaneously Enoxaparin 1mg / kg SQ daily is also taken to prevent thrombosis. Patients' blood pressure, along with other vital signs, is checked regularly and at regular intervals. Standard drug treatment according to the treatment protocols of the National Committee for COVID-19 includes Hydroxychloroquine / Chloroquine Phosphate:• Hydroxychloroquine sulfate tablets 200 mg or chloroquine phosphate tablets 250 mg (equivalent to 150 mg base dose) 2 tablets every 12 hours on the first day and then one tablet every 12 hours for at least 7 days and up to 14 days. One of the following medications at the discretion and diagnosis of the treating physician:• Caltrate tablets (Lopinavir / Ritonavir) 50/200 mg every 12 hours 2 pieces after meals for at least 7 days and a maximum of 14 days• Tablets (Atazanavir / Ritonavir) 300/100 One tablet daily with food or Atazanavir 400 mg daily for at least 7 days and up to 14 days
Category

Treatment - Drugs

2

Description

Control group: Group B: The standard drug treatment is based on the treatment protocols of the National Committee for COVID-19 and the placebo(distilled water) is given as a subcutaneous injection five times a day for 5 days. Standard drug treatment according to the treatment protocols of the National Committee for COVID-19 includes Hydroxychloroquine / Chloroquine Phosphate:• Hydroxychloroquine sulfate tablets 200 mg or chloroquine phosphate tablets 250 mg (equivalent to 150 mg base dose) 2 tablets every 12 hours on the first day and then one tablet every 12 hours for at least 7 days and up to 14 days. One of the following medications at the discretion and diagnosis of the treating physician:• Caltrate tablets (Lopinavir / Ritonavir) 50/200 mg every 12 hours 2 pieces after meals for at least 7 days and a maximum of 14 days• Tablets (Atazanavir / Ritonavir) 300/100 One tablet daily with food or Atazanavir 400 mg daily for at least 7 days and up to 14 days

Category

Placebo

Recruitment centers

1

Recruitment center

Name of recruitment center

Shahid Mohammadi Hospital

Full name of responsible person

Dr. Mehdi Hassaniazad

Street address

Boulevard of the Islamic Republic of Iran, Bandar Abbas

City

Bandar Abbas

Province

Hormozgan

Postal code

7916613885

Phone

+98 76 3334 7000

Fax

+98 76 3334 5003

Email

shmh@hums.ac.ir

Web page address

https://shmh.hums.ac.ir/

Sponsors / Funding sources

1

Sponsor

Name of organization / entity

Bandare-abbas University of Medical Sciences

Full name of responsible person

Dr. Teamur Aghamolaei

Street address

Deputy of Research and Technology, Hormozgan University of Medical Sciences, Shahid Chamran Boulevard, Bandar Abbas, Hormozgan, Iran

City

Bandar Abbas

Province

Hormozgan

Postal code

7916613885

Phone

+98 76 3333 7192

Fax

+98 76 3333 7192

Email

research@hums.ac.ir

Web page address

https://resv.hums.ac.ir/

Grant name

Grant code / Reference number

Is the source of funding the same sponsor organization/entity?

Yes

Title of funding source

Bandare-abbas University of Medical Sciences

Proportion provided by this source

100

Public or private sector

Public

Domestic or foreign origin

Domestic

Category of foreign source of funding

empty

Country of origin

Type of organization providing the funding

Academic

Person responsible for general inquiries

Contact

Name of organization / entity

Bandare-abbas University of Medical Sciences

Full name of responsible person

Dariush Hooshyar

Position

Student of Medicine

Latest degree

Medical doctor

Other areas of specialty/work

General Practitioner

Street address

Faculty of Medicine, Pardis unit of Hormozgan University of Medical Sciences, End of Imam Hossein Blvd., Bandar Abbas, Iran

City

Bandar Abbas

Province

Hormozgan

Postal code

7919693116

Phone

+98 990 038 7226

Email
dariush.hooshyar@gmail.com

Person responsible for scientific inquiries

Contact
Name of organization / entity
Bandare-abbas University of Medical Sciences
Full name of responsible person
Dr. Mitra Kazemijahromi
Position
Assistant Professor
Latest degree
Subspecialist
Other areas of specialty/work
Internal Medicine
Street address
Office of the Subspecialty Internal Group, Shahid Mohammadi Hospital, Boulevard of the Islamic Republic of Iran, Bandar Abbas
City
Bandar Abbas
Province
Hormozgan
Postal code
7916613885
Phone
+98 917 791 2820
Email
mitra.kazemijahromi@gmail.com

Person responsible for updating data

Contact
Name of organization / entity
Bandare-abbas University of Medical Sciences
Full name of responsible person
Dr. Mitra Kazemijahromi
Position
Assistant Professor
Latest degree
Subspecialist
Other areas of specialty/work
Internal Medicine
Street address
Office of the Subspecialty Internal Group, Shahid Mohammadi Hospital, Boulevard of the Islamic Republic of Iran, Bandar Abbas
City
Bandar Abbas
Province
Hormozgan
Postal code
7916613885
Phone
+98 917 791 2820
Email
mitra.kazemijahromi@gmail.com

Sharing plan

Deidentified Individual Participant Data Set (IPD)
Undecided - It is not yet known if there will be a plan to make this available
Study Protocol
Undecided - It is not yet known if there will be a plan to make this available
Statistical Analysis Plan
Undecided - It is not yet known if there will be a plan to make this available
Informed Consent Form
Undecided - It is not yet known if there will be a plan to make this available
Clinical Study Report
Undecided - It is not yet known if there will be a plan to make this available
Analytic Code
Undecided - It is not yet known if there will be a plan to make this available
Data Dictionary
Undecided - It is not yet known if there will be a plan to make this available
